# Beyond biogeographic patterns: Processes shaping the microbial landscape in soils and sediments along the Yangtze River

**DOI:** 10.1002/mlf2.12062

**Published:** 2023-03-26

**Authors:** Wenjie Wan, Geoffrey M. Gadd, Ji‐Dong Gu, Wenzhi Liu, Peng Chen, Quanfa Zhang, Yuyi Yang

**Affiliations:** ^1^ Key Laboratory of Aquatic Botany and Watershed Ecology Wuhan Botanical Garden Chinese Academy of Sciences Wuhan China; ^2^ Danjiangkou Wetland Ecosystem Field Scientific Observation and Research Station Chinese Academy of Sciences & Hubei Province Wuhan China; ^3^ Geomicrobiology Group, School of Life Sciences University of Dundee Dundee Scotland UK; ^4^ State Key Laboratory of Heavy Oil Processing, State Key Laboratory of Petroleum Pollution Control China University of Petroleum Beijing China; ^5^ Environmental Science and Engineering Group Guangdong Technion‐Israel Institute of Technology Guangdong China

**Keywords:** environmental breadth, environmental constraint, phylogenetic signal, species replacement, stochasticity versus determinism

## Abstract

Deciphering biogeographic patterns of microorganisms is important for evaluating the maintenance of microbial diversity with respect to the ecosystem functions they drives. However, ecological processes shaping distribution patterns of microorganisms across large spatial‐scale watersheds remain largely unknown. Using Illumina sequencing and multiple statistical methods, we characterized distribution patterns and maintenance diversity of microorganisms (i.e., archaea, bacteria, and fungi) in soils and sediments along the Yangtze River. Distinct microbial distribution patterns were found between soils and sediments, and microbial community similarity significantly decreased with increasing geographical distance. Physicochemical properties showed a larger effect on microbial community composition than geospatial and climatic factors. Archaea and fungi displayed stronger species replacements and weaker environmental constraints in soils than that in sediments, but opposite for bacteria. Archaea, bacteria, and fungi in soils showed broader environmental breadths and stronger phylogenetic signals compared to those in sediments, suggesting stronger environmental adaptation. Stochasticity dominated community assemblies of archaea and fungi in soils and sediments, whereas determinism dominated bacterial community assembly. Our results have therefore highlighted distinct microbial distribution patterns and diversity maintenance mechanisms between soils and sediments, and emphasized important roles of species replacement, environmental adaptability, and ecological assembly processes on microbial landscape. Our findings are helpful in predicting loss of microbial diversity in the Yangtze River Basin, and might assist the establishment of environmental policies for protecting fragile watersheds.

## Impact statement

Understanding biogeographic patterns and diversity maintenance mechanisms of microorganisms is important to evaluate and predict diversity‐driven ecosystem functions. Our DNA‐based datasets and statistical analyses have revealed distribution patterns and diversity maintenance of microorganisms (i.e., archaea, bacteria, and fungi) in soils and sediments along the Yangtze River. Ecological indicators including species replacement, environmental breadths, phylogenetic signals, and community assembly processes revealed mechanisms underlying the different microbial landscapes between soils and sediments. Our findings extend knowledge of the generation and maintenance of microbial diversity in watershed ecosystems, and are helpful in predicting microbial diversity loss and guiding ecological protection strategies for vulnerable watersheds.

## INTRODUCTION

Watershed ecosystems function in maintaining biodiversity, supplying aquatic products, and facilitating economic development^1^. However, watershed ecosystems are increasingly threatened by disasters under the scenario of global climate change (e.g., flooding and extreme temperatures) and potentially harmful anthropogenic activities (e.g., excessive agricultural fertilization and wastewater discharge)^2^, ^3^, ^4^, which can result in soil erosion, channel siltation, and water pollution. Evaluating the health and potential of watershed ecosystems by assessing water quality and land use type (e.g., farmland and woodland) is especially challenging when watershed ecosystems are under increasing pressure from human society and development^5^, ^6^. Microorganisms mediate key element transformation (e.g., carbon decomposition, nitrogen fixation, and phosphorus mineralization)^7^, ^8^, and are important components of all food webs^9^. Microbial diversity is therefore regarded as a bioindicator for ecosystem functions^10^, ^11^, and provides a means of appraising the ecological health of a watershed ecosystem^2^, ^12^. Therefore, it is important to elucidate distribution patterns and diversity maintenance mechanisms of microorganisms in watershed ecosystems.

Deciphering biogeographic distribution is one of the fundamental themes in community ecology^13^. The biogeographic distribution of microorganisms is constrained by geospatial variations and local environmental heterogeneity^14^, ^15^, ^16^, which makes the relationships between microbial diversity and environmental variables extremely complex. Clarifying the relationship of microbial diversity with the environment is fundamentally important to provide predictive understanding of microbial diversity‐driven ecosystem processes and functions. Some ecological theories that attempt to predict diversity‐environment linkage mainly consider nutrient availability and environmental stress factors^17^, ^18^. The maintenance mechanisms of microbial diversity can be clarified by environmental adaptability and ecological assembly processes^15^, ^18^, ^19^. Environmental adaptability reflects the resistance of species in response to environmental change by evaluating their environmental breadth and phylogenetic signal^11^, ^20^, ^21^. For instance, a prior study reports that bacterial community displays stronger environmental adaptability in the nutrient‐rich conditions of a eutrophic lake ecosystem, showing broader environmental breadths and stronger phylogenetic signals^22^. Ecological community assembly, which determines microbial community composition and coexistence as well as the community function they provide, involves stochastic and deterministic processes^23^. Deterministic processes (i.e., sorting), which comprise ecological selection imposed by biotic effects and environmental filtering, affect species‐environment fitness and therefore shape the relative abundance and community composition of various species^24^. Stochastic processes arising from random events lead to many species occurring in identical or highly coincident niches^24^. Therefore, deciphering microbial community in response to abiotic factors is essential for a better understanding of microbial diversity maintenance in different habitats. However, microbial environmental adaptations and ecological assembly processes have not been simultaneously studied in soils and sediments in watershed ecosystems.

Soils and sediments are regarded as nutrient sinks and microbial habitats in watershed ecosystems, providing nutrition for primary producers, underpinning food webs, and maintaining ecological functions^25^, ^26^, ^27^, ^28^. Grass‐covered soil and adjacent near‐shore sediment are two notably different environments and undergo differing environmental and ecological events, including soil erosion, water discharge, and sediment scouring and exposure. This stimulated our interest in investigating differences in abiotic (i.e., physicochemical properties) and biotic (e.g., microbial distribution patterns) properties between soils and sediments in a watershed ecosystem. For this reason, we chose the Yangtze River Basin as our research site and collected grass‐covered soil and adjacent near‐shore sediment samples (Table S1 and Figure S1). In this research, we aimed to (i) investigate distribution patterns of the microorganisms (i.e., archaea, bacteria, and fungi) and disentangle the maintenance mechanisms of microbial diversity, and (ii) evaluate differences in biotic properties between soils and sediments of the Yangtze River. Given that nutrient elements (e.g., carbon, nitrogen, phosphorus, and sulfur) are more abundant in soils than in sediments (Figure S2), we hypothesized that stochastic processes would predominantly affect community assemblies of microorganisms in soils rather than in sediments. Illumina MiSeq sequencing and measurements physicochemical properties of soil and sediment were carried out to collect the necessary data to validate our hypothesis via multiple statistical analyses. We found clear divergences in microbial distribution patterns, environmental adaptation, and ecological assembly processes between soils and sediments.

## RESULTS

### General distribution patterns of microorganisms in soils and sediments

**Figure 1:**
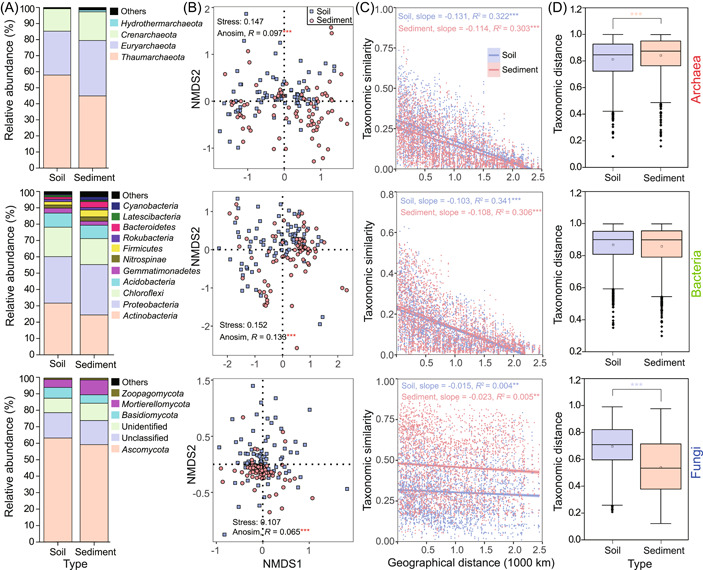
Community composition of archaea, bacteria, and fungi in soils and sediments. (A) Stacked columns reflect relative abundances (>1%) of microbial phyla. (B) NMDS plots display microbial community similarity between soils and sediments. (C) Distance‐decay curves show microbial taxonomic similarity based on Bray–Curtis similarity against geographical distance. (D) Box plots exhibit difference in microbial taxonomic distance based on Bray–Curtis dissimilarity between within soils and within sediments. Asterisks (B–D) denote significance (**p < 0.01; ***p < 0.001), and blue and red asterisks reflect larger value for soil and sediment, respectively. NMDS, nonmetric multidimensional scaling.

A total of 13,352 archaeal amplicon sequence variants (ASVs) were found in soils and sediments, and they shared 7292 ASVs (Figure S3). Similarly, bacterial and fungal communities had 19,929 and 13,557 ASVs, respectively, and they separately shared 14,710 and 4318 ASVs between soils and sediments (Figure S3). Archaeal ASVs were mainly identified as *Thaumarchaeota* (57.89% for soils; 44.89% for sediments), *Euryarchaeota* (27.33% for soils; 34.54% for sediments), and *Crenarchaeota* (14.02% for soils; 17.89% for sediments) (Figure 1A). Bacterial ASVs were mainly identified as *Actinobacteria* (31.67% for soils; 24.42% for sediments), *Proteobacteria* (28.39% for soils; 30.82% for sediments), and *Chloroflexi* (18.05% for soils; 15.90% for sediments) (Figure 1A). Fungal ASVs were mainly identified as *Ascomycota* (63.19% for soils; 59.18% for sediments), *Basidiomycota* (6.49% for soils; 5.17% for sediments), and *Mortierellomycota* (4.83% for soils; 8.95% for sediments) (Figure 1A). Nonmetric multidimensional scaling (NMDS) plots showed distinct differences in the community composition of archaea, bacteria, and fungi (Figure 1B), and pairwise analyses of similarity (ANOSIM) results confirmed significant differences (for archaea, *R* = 0.097, *p* < 0.001; for bacteria, *R* = 0.133, *p* < 0.001; for fungi, *R* = 0.065, *p* < 0.001). Significant distance‐decay relationships (DDRs) were found for community similarities of archaea (*p* < 0.001), bacteria (*p* < 0.001), and fungi (*p* < 0.01) in soils and sediments (Figure 1C). However, the fitness was quite low for fungi in soils and sediments (*R* ^2^ < 0.1, Figure 1C), suggesting a weak decay of community similarity with an increase in geographic distance. Significantly larger compositional dissimilarity was found for archaea in sediments and fungi in soils than for archaea in soils and fungi in sediments, respectively (*p* < 0.001; Figure 1D). Slightly larger compositional dissimilarity was found for bacteria in soils (*p* > 0.05). These results indicated that there were distinct differences in the geographical distribution of microorganisms between soils and sediments.

**Figure 2:**
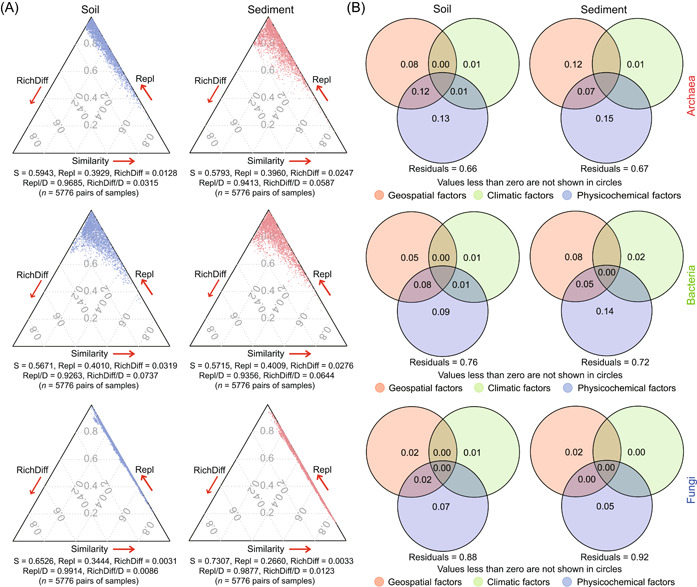
Taxonomic β‐diversity decomposition and environmental factors affecting microbial community composition. (A) Triangular plots show effects of species replacement and richness difference on microbial taxonomic β‐diversity. Each point represents a pair of samples. Its position is determined by a triplet of values from the S = (1 − D) (S, similarity; D, dissimilarity), Repl (species replacement), and RichDiff (richness difference) matrices. (B) VPA shows effects of geospatial, climatic, and physicochemical factors on microbial community composition. VPA, variation partitioning analysis.

We found that species replacement and richness difference showed different effects on microbial compositional dissimilarities through disassembling microbial taxonomic β‐diversities (Figure 2A). Species replacement showed comparably larger effects than richness difference for soils and sediments. Archaea (soils, 0.9685; sediments, 0.9413) and fungi (soils, 0.9914; sediments, 0.9877) displayed relatively large species replacement (species replacement/dissimilarity) in soils, whereas bacteria showed a relatively large species replacement in sediments (soils, 0.9263; sediments, 0.9356). These results indicate that archaea and fungi were more environmentally constrained in sediments, and bacteria were more environmentally constrained in soils. According to results of variation partitioning analysis (VPA), local physicochemical properties showed larger effects on microbial (i.e., archaea, bacteria, and fungi) community composition than geospatial and climatic factors (Figure 2B). The environmental factors (i.e., physicochemical, geospatial, and climatic factors) explained relatively more compositional variations in archaeal and fungal communities in soils as well as the bacterial community in sediments. According to results of permutational multivariate analysis of variance (PERMANOVA) (Table S2), environmental factors (e.g., longitude, pH, and available iron) showed different effects on microbial community composition.

Significantly higher fungal diversity, represented by Shannon–Wiener index, was found for soils than for sediments (*p* < 0.01; Figure S4). Community diversities of archaea and bacteria were slightly higher for sediments than for soils (*p* > 0.05). According to results of Pearson's correlations (Table S3), community diversities of microorganisms (i.e., archaea, bacteria, and fungi) in soils and sediments were significantly correlated with longitude (*p* < 0.05 or *p* < 0.01 or *p* < 0.001).

### Microbial environmental adaptability at taxonomic and phylogenetic levels

**Figure 3:**
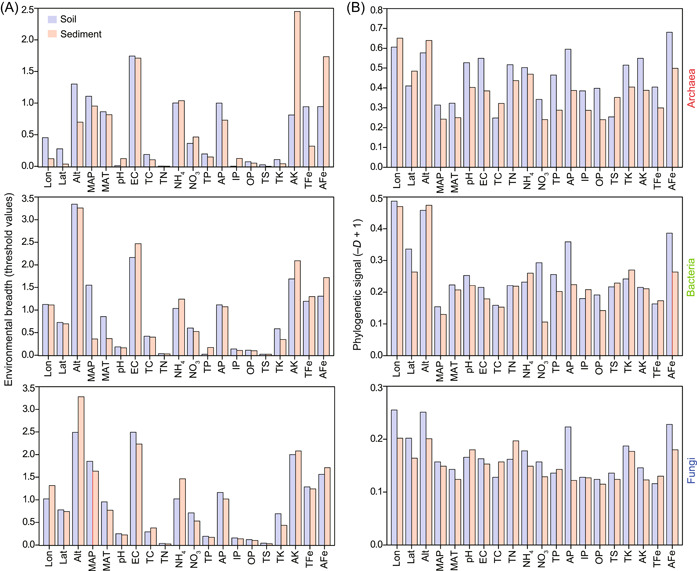
Environmental adaptability of microorganisms in soils and sediments. (A) Environmental breadths of microorganisms reflect microbial environmental adaptation in relation to taxonomic diversity by calculating threshold values in response to each tested environmental factor. The threshold values were standardized using log_10_ (original threshold value + 1). (B) Phylogenetic signals of microorganisms reveal phylogenetic conservatism for environmental preferences by computing Fritz–Purvis *D* values. AFe, available iron; AK, available potassium; Alt, altitude; AP, available phosphorus; EC, electrical conductivity; IP, inorganic phosphorus; Lat, latitude; Lon, longitude; MAP, mean annual precipitation; MAT, mean annual temperature; NH_4_, ammonium nitrogen; NO_3_, nitrate nitrogen; OP, organic phosphorus; TC, total carbon; TFe, total iron; TK, total potassium; TN, total nitrogen; TP, total phosphorus; TS, total sulfur.

Microorganisms (i.e., archaea, bacteria, and fungi) showed wider ranges of environmental thresholds for most environmental variables (numbers of environmental factors showing broader environmental breadths/20 environmental factors) in soils (70% for archaea; 65% for bacteria; 70% for fungi) than those in sediments (30% for archaea; 35% for bacteria; 30% for fungi) (Figure 3A and Table S4). These results indicate that archaea, bacteria, and fungi might display stronger environmental adaptability in soils than in sediments at the taxonomic level. Microorganisms (i.e., archaea, bacteria, and fungi) displayed stronger phylogenetic signals for most environmental variables (numbers of environmental factors showing stronger phylogenetic signals/20 environmental factors) in soils (75% for archaea; 70% for bacteria; 75% for fungi) than those in sediments (25% for archaea; 30% for bacteria; 25% for fungi) (Figure 3B and Table S4). This implies that archaea, bacteria, and fungi display stronger environmental adaptation in soils than in sediments at the phylogenetic level.

### Ecological assembly processes and environmental constraints

**Figure 4:**
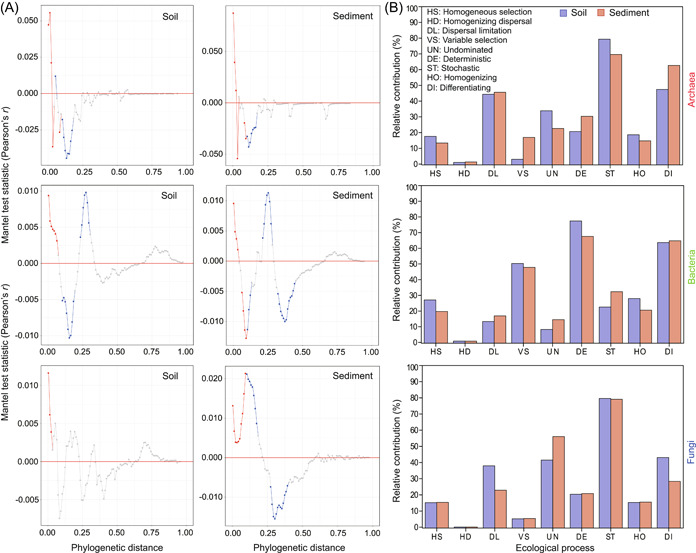
Phylogenetic features and community assemblies of microorganisms in soils and sediments. (A) Mantel correlograms show significant phylogenetic signals across short phylogenetic distances. Each point indicates the Mantel correlation coefficient of each given range of phylogenetic distances. Red, blue, and gray symbols represent highly significant (*p* < 0.01), significant (*p* < 0.05), and non‐significant (*p* > 0.05) correlations, respectively. (B) Relative contributions of ecological processes to community assemblies of microorganisms based on null model analysis. Differentiating = variable selection + dispersal limitation; Homogenizing = homogeneous selection + homogenizing dispersal.

Mantel correlograms consistently displayed significant positive correlations across short phylogenetic distances for archaea, bacteria, and fungi in soils and sediments (Figure 4A). For nearly all groups (except for fungi in soils), we also found notably negative correlations and nonsignificant correlations. These results indicated that significant phylogenetic signals across relatively short phylogenetic distances were found for microbial communities along environmental gradient.

**Figure 5:**
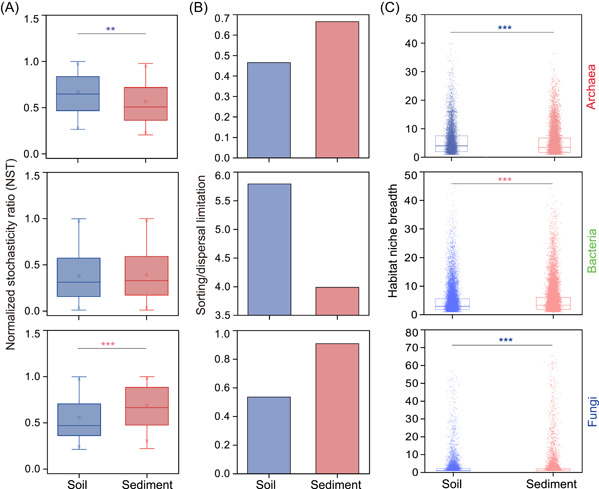
Stochastic community assembly and environmental constraints of microorganisms in soils and sediments. (A) Normalized stochasticity ratio (NST) index shows effect of stochasticity on microbial community assembly. When NST index is above the boundary of 0.5, it represents microbial community is more stochastic assembly. (B) A large ratio of sorting to dispersal limitation based on null model analysis reflects strongly environmental constraint. (C) Broad niche breadth reveals a more flexible metabolism and in turn denotes weakly environmental restrict. Asterisks denote significance (**p < 0.01; ***p < 0.001).

Null model‐based ecological processes showed different contributions to community assemblies of the archaea, bacteria, and fungi in soils and sediments (Figure 4B). Dispersal limitation (44.37% for soils; 45.65% for sediments), stochastic processes (79.33% for soils; 69.61% for sediments), and differentiating processes (47.45% for soils; 62.67% for sediments) showed main effects on archaeal community assembly. In contrast, variable selection (50.29% for soils; 47.89% for sediments), deterministic processes (77.40% for soils; 67.62% for sediments), and differentiating processes (63.65% for soils; 64.81% for sediments) displayed main influences on bacterial community assembly. Homogeneous selection, homogenizing dispersal, “undominated” processes, and homogenizing processes showed limited impacts on community assemblies of archaea and bacteria. However, dispersal limitation (37.94% for soils; 22.93% for sediments), and “undominated” processes (41.56% for soils; 56.07% for sediments) showed more effects on fungal community assembly for soils and sediments. Consequently, stochastic processes (79.65% for soils; 79.15% for sediments) and differentiating processes (43.11% for soils; 28.39% for sediments) showed main effects on fungal community assembly (Figure 4B). Additionally, the normalized stochasticity ratio revealed that community assemblies of archaea and fungi were stochasticity‐dominated in both soils and sediments, whereas community assemblies of bacteria were more determinism‐dominated in both soils and sediments (Figure 5A).

According to results of a null model, the ratios of sorting/dispersal limitation were relatively high for archaeal and fungal communities in sediments rather than soils, but the opposite was found for the bacterial community (Figure 5B). Based on analysis of habitat niche breadth, soil archaea and fungi displayed more flexible metabolic potential than corresponding sediment archaea and fungi. However, the opposite occurred for the bacterial community (Figure 5C). These results therefore indicated that archaea and fungi were more environmentally constrained in sediments, whereas bacteria were more environmentally restricted in soils.

## DISCUSSION

Protecting watershed ecosystems by environmentally friendly and resource‐saving procedures is a timely concern. Typically, trees and grasses are planted along riverbanks to prevent soil loss. Simultaneously, dredging is implemented to deepen channels to enhance water flow. Following the implementation of the “Yangtze River Protection Law” on March 1, 2021, we were inspired to evaluate the ecological health of the Yangtze River by evaluating differences in distribution patterns and diversity maintenance mechanisms of microorganisms (i.e., archaea, bacteria, and fungi) between soils and sediments. We found distinct differences in distribution patterns, environmental adaptability, ecological assembly processes, and environmental constraints of microorganisms between soils and sediments.

### Distinct microbial distribution patterns between soils and sediments

Community similarity of microorganisms (i.e., archaea, bacteria, and fungi) in soils and sediments decayed with an increase in geographical distance, which is similar to prior findings for both terrestrial and aquatic ecosystems^15^, ^19^, ^29^. Ecological processes (e.g., selection, drift, dispersal, and/or mutation) drive microbial biogeography^30^. Differentiating processes showed large effects on microbial community assembly, which led to community compositional dissimilarity and therefore significant DDRs. We found distinct distribution patterns of microorganisms between soils and sediments. This phenomenon might arise primarily from differences in environmental heterogeneity between soils and sediments (Figure S2). Unlike the pH driven bacterial geographic distribution in forest soils from eastern China^31^ and British soils^32^, longitude was closely linked with diversity and community composition of archaea, bacteria, and fungi in both soils and sediments. The decisive role of longitude could be primarily due to the close linkage between longitude and other physicochemical factors (e.g., pH, total carbon, and available phosphorus; Table S5).

Species replacement rather than richness abundance were responsible for community compositional dissimilarity. Species replacement depends on the migration potential of the microbial community and external environmental disturbances^33^, ^34^. Microbial migration potential subjects to individual dispersal capability and environmental filtering^17^. Strong environmental disturbance to microbial communities occurs in the Yangtze River Basin during the rain season and flood season, which results in nutrient fluctuations and ecological drift^18^. Therefore, species replacement partly contributed to community compositional discrepancy for the microorganisms. Notable differences in distribution patterns of microorganisms between soils and sediments suggested that there was no vigorous exchange between soils and sediments, which further confirms the ecological importance of planting grasses and/or trees in watershed ecosystems.

### Stronger environmental adaptability of microorganisms in soils rather than sediments

Most studies have investigated the activity, abundance, diversity, and structure of microbial communities in watershed ecosystems^35^, ^36^ with little reference to environmental adaptability. We have attempted to estimate differences in archaeal, bacterial, and fungal communities between soils and sediments in response to tested environmental factors in the Yangtze River. We found that microbial environmental adaptability was stronger for soils than for sediments at both taxonomic and phylogenetic levels.

Environmental breadth and phylogenetic signal approaches are useful for estimating microbial environmental adaptability^21^, ^37^. For instance, relative abundances of *Agrobacteria*, *Cytophaga*, *Dysgonomonas*, and *Nesterenkonia* spp. increase at low levels of total phosphorus, whereas relative abundances of *Clavibacter*, *Cloacibacterium*, and *Sulfurimonas* spp. increase at high levels of total phosphorus in rivers and streams along the Oklahoma–Arkansas border^37^. Additionally, microbial responses to environmental variables display phylogenetic conservatism at different taxonomic levels^38^. A 144 *Bacillus* sp. isolated from a sediment display deep phylogenetic signals for biofilm formation, mobility, and prototrophy according to Fritz–Purvis *D* tests^39^. In this study, microorganisms in soils possessed broader environmental breadths and stronger phylogenetic signals than those from sediments. This phenomenon is similar to the findings for two different habitats. For example, functional organic phosphorus‐mineralizing‐related bacteria exhibit broader environmental breadths and stronger phylogenetic signals at high altitudes (>1500 m) than at low elevations (<1500 m) in the Shennongjia virgin forest^40^. The divergence in microbial environmental adaptability between these two habitats (e.g., soils and sediments) might be due to differences in nutrient availability and microbial resistance to environmental change. Most tested nutrients were more abundant in soils rather than in sediments (Figure S2), and it has been reported that nutrient deficiency can intensify microbial competition and some microorganisms lose their opportunity for survival^41^. Additionally, water scouring exacerbates nutrient loss. Aquatic microorganisms are relatively easy to disperse through water flow, and thus aquatic predators (e.g., protozoa and bacteria) can easily capture their corresponding prey^42^, ^43^. The soils analyzed in this study were covered by grass, which protects soil microorganisms from deleterious effects of intense radiation. A prior study reports that ultraviolet radiation can damage microbial DNA in the environment^44^. Such findings mentioned above might explain why soil microorganisms showed stronger environmental adaptability than those in sediments.

### Different ecological processes mediating microbial community assembly

Stochastic and/or deterministic processes dominated microbial (i.e., archaea, bacteria, and fungi) community assemblies in different environments (e.g., soils, sediments, and water)^8^, ^15^, ^18^. For instance, archaeal and bacterial community assemblies are stochasticity‐dominated in subtropical mangrove sediments^8^. Stochastic processes dominate community assembly of soil fungi across 29 lake islands^45^. Unexpectedly, stochastic and deterministic processes separately dominated archaeal and bacterial community assemblies. However, both stochastic and deterministic processes affected fungal community assembly. Some divergences might be attributable to geography^14^. Some literature has reported that geospatial and climatic factors show noticeable effects on microbial community assembly^40^, ^46^, ^47^. Previous studies have reported that nutrient availability affects microbial community assembly^18^, ^23^. Stochastic processes contribute the most to microbial community assemblies in nutrient‐rich environments, whereas deterministic processes tend to dominate microbial community assemblies in nutrient‐poor environments^48^. In addition, different ecological processes dominating community assemblies of archaea, bacteria, and fungi might be also due to organismal lifestyle and cell size^49^, ^50^. Archaea and bacteria are typically regarded as unicellular, whereas most fungi are typically characterized as filamentous, producing extreme branching mycelia^51^. Filamentous fungi are much larger compared to archaea and bacteria, and therefore less easily disperse in the vegetative state. An earlier study has also reported that relative contributions of environmental selection and neutral processes to microbial community assembly directly depends on body size^50^.

Archaea and fungi were more environmentally constrained in sediments, whereas bacteria were more environmentally restricted in soils. This discrepancy in environmentally constraint might arise mainly from differences in nutrient levels and gas circulation between soils and sediments. Microorganisms are clearly sensitive to gas flow and nutrient availability^52^, ^53^, which affects growth and metabolism and therefore determines their environmental constraints. Unexpectedly, we found that the environmental constraint of microorganisms had close linkage with species replacement in the Yangtze River watershed. This phenomenon is reasonable because a strong species replacement implies a strong dispersal potential for the microorganisms and/or weak environmental filtering^18^, ^34^, which in turn reflects weak environmental constraints of microorganisms. Further studies are needed to verify these findings in other different watershed ecosystems to allow for better generalization.

**Figure 6:**
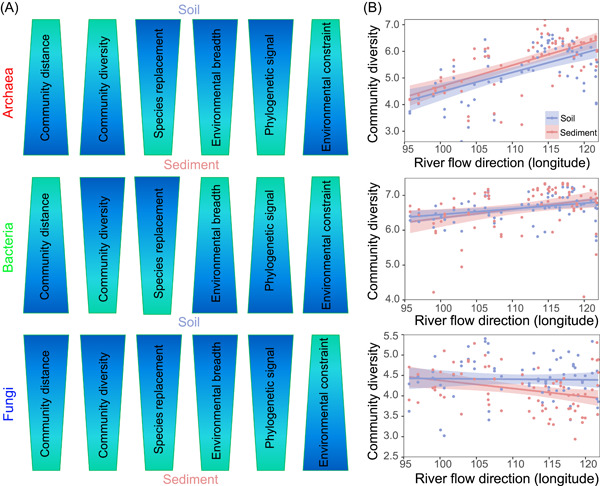
A conceptual model summarizing differences in microbial communities between soils and sediments. (A) Differences in ecological responses of microorganisms between soils and sediments. Community distance and community diversity are represented by Bray–Curtis dissimilarity and the Shannon–Wiener index, respectively. Species replacement, environmental breadth, and phylogenetic signal are revealed by species replacement/dissimilarity, threshold analysis, and Fritz–Purvis *D* test, respectively. Environmental constraints of microorganisms are simultaneously reflected by sorting/dispersal limitation and habitat niche breadth. (B) Relationships between river flow direction and community diversity in soils and sediments.

Ultimately, a conceptual paradigm was constructed to summarize the findings for the Yangtze River watershed in terms of microbial ecology (Figure 6). Broader environmental breadths and stronger phylogenetic signals of microorganisms (i.e., archaea, bacteria, and fungi) were found for soils rather than sediments (Figure 6A). However, microbial community distance, community diversity, species replacement, and environmental constraint showed inconsistent trends for soils and sediments. Considering the importance of microbial diversity and the decisive role of longitude, we have described the linkage between microbial diversity and longitude (Figure 6B). Community diversities of archaea and bacteria significantly increased along river flow direction, whereas fungal diversity notably decreased along river flow direction. Considering the increasing population and developing economy activity in eastern China, excessive human activities will affect and even result in loss of microbial diversity. A meta‐analysis has reported that afforestation could significantly enhance fungal diversity and slightly promote bacterial diversity^54^. Ecological protection measures, including planting trees and other vegetation for preventing soil erosion and reducing pollutant discharge (e.g., toxic metals and xenobiotics), should be jointly employed to maintain and enhance the health and potential of watershed ecosystems.

In conclusion, we demonstrate distinct distribution patterns and diversity maintenance mechanisms of microorganisms (i.e., archaea, bacteria, and fungi) occurring between soils and sediments along the Yangtze River. Longitude as a geospatial factor shows close linkages with diversity and community composition of microorganisms. Our findings have deciphered significant differences in abiotic and biotic properties between soils and sediments along the Yangtze River, and might be helpful to evaluate watershed health and predict diversity loss under the background of global climate change. Considering the important ecological significance of microbial diversity, experimental validation should be further implemented by studies of more different watershed systems.

## MATERIALS AND METHODS

### Sample collection and environmental parameters

A total of 38 sites along the Yangtze River were selected for sampling between October 9th and November 19th in 2019 (Table S1 and Figure S1). Grass‐covered soils and adjacent near‐shore sediments were selected (see sampling details in Figure S1), and five soil or sediment cores were collected at a depth of 0–20 cm with a hand core probe at each site. Soil or sediment cores were then mixed evenly to form a composite soil or sediment sample. Each site had two replicate soils and sediments, and a total of 76 soil samples and 76 sediment samples were collected for experiments. Simultaneously, approximately 10 g of soils or sediments from each sample were placed in a sterile tube and then immediately stored in a portable refrigerator at −18°C for later DNA extraction^19^. The remaining soil and sediment samples were covered with dry ice and transported to the laboratory within 48 h.

Geospatial information of longitude (Lon), latitude (Lat), and altitude (Alt) was recorded for each site, and climatic properties of mean annual precipitation (MAP) and mean annual temperature (MAT) were gained from the WorldClim database (https://www.worldclim.org) (Table S1). We determined physicochemical properties of soils and sediments according to standard protocols. The physicochemical factors included pH, electrical conductivity (EC), total carbon (TC), total nitrogen (TN), ammonium nitrogen (NH_4_), nitrate nitrogen (NO_3_), total phosphorus (TP), available phosphorus (AP), inorganic phosphorus (IP), organic phosphorus (OP), total sulfur (TS), total potassium (TK), available potassium (AK), total iron (TFe), and available iron (AFe). Descriptions of the measurement of physicochemical properties of soils and sediments are provided in Supporting Information: Supplementary Method 1.

### DNA extraction, amplicon sequencing and processing

Genomic DNA was extracted from soils or sediments according to the ISO‐11063 standardized DNA extraction method^55^. Universal primers 524F (5′‐TGY CAG CCG CCG CGG TAA‐3′) and 958R (5′‐YCC GGC GTT GAV TCC AAT T‐3′) were employed to amplify the archaeal 16S ribosomal RNA (rRNA) gene^56^. Universal primers 338F (5′‐ACT CCT ACG GGA GGC AGC A‐3′) and 806R (5′‐GGA CTA CHV GGGTWT CTA AT‐3′) were used to amplify the bacterial 16S rRNA gene^57^. Universal primers ITS1 (5′‐GGA AGT AAA AGT CGT AAC AAG G‐3′) and ITS2 (5′‐GCT GCG TTC TTC ATC GAT GC‐3′) were applied to amplify the fungal ITS^58^. Sequencing was performed on the Illumina MiSeq platform at the Personal Biotechnology Co., Ltd. The raw sequences were run through the QIIME2 pipeline to obtain denoised, chimera‐free, nonsingleton ASVs^59^.

### Data analyses

We eliminated ASVs that contained less than 20 reads in 152 samples to avoid random effects on the identification of microbial taxa. Significant differences in the data, if not otherwise stated, were analyzed by using the Wilcoxon rank‐sum test when the data did not follow a normal distribution. We applied Venn diagrams, NMDS plots, and ANOSIM to show microbial compositional differences between soils and sediments. The PERMANOVA was used to estimate effects of different environmental variables on microbial community composition. The DDRs were estimated by computing the slope of an ordinary least‐square regression between geographical distance and taxonomic similarity (1−Bray‐Curtis distance). The β‐diversities of microbial communities were decomposed by species replacement and richness difference^34^. The VPA was used to explore effects of geospatial factors, climatic factors, and physicochemical factors on microbial community composition.

We used threshold indicator taxa analysis (TITAN) to reveal the environmental breadth of microorganisms (i.e., archaea, bacteria, and fungi) in response to each tested environmental variable^20^. Trait information of microorganisms, reflecting microbial preference for a given environmental variable, was evaluated by calculating Spearman correlations between relative abundances of ASVs and environmental factors^11^. The Fritz–Purvis *D* test was used to reflect phylogenetic signals of microorganisms in response to each tested environmental factor by comparing the sister clade divergences in the trait against those expected for a random phylogenetic pattern^21^. The evolution of a tested trait (i) is more conserved than expected by chance when –*D* + 1 > 0 or (ii) does not denote a strong signal when –*D* + 1 = 0^21^. Detailed descriptions of estimation of environmental breadths and phylogenetic signals are reported previously^19^, ^20^, ^21^, and are also summarized in Supporting Information: Supplementary Method 2. Higher percentage, that is, numbers of environmental factors showing broader environmental breadths or stronger phylogenetic signals/20 environmental factors × 100%, was used to reflect whether microorganisms had stronger environmental adaptation in soils or sediments^22^, ^23^.

Mantel correlograms were used to reveal whether a significantly phylogenetic signal from the microbial community along an environmental gradient occurred at a short phylogenetic distance^60^. According to the null model, we calculated the β‐net Relatedness Index (βNRI) and Bray–Curtis‐based Raup–Crick (RC_bray_) index to estimate relative contributions of ecological processes^61^. Theses ecological processes included variable selection (βNRI > 1.96), homogeneous selection (βNRI < −1.96), dispersal limitation (│βNRI│ < 1.96 and RC_bray_ > 0.95), homogenizing dispersal (│βNRI│ < 1.96 and RC_bray_ < −0.95), “undominated” processes (│βNRI│ < 1.96 and │RC_bray_│ < 0.95), deterministic processes (│βNRI│>1.96), and stochastic processes (│βNRI│ < 1.96). The NST was used to further reveal the contribution of a stochasticity to microbial community assembly^62^. The Levins’ niche breadth index was used to reflect metabolic flexibility of microbial community^15^.

Statistical analyses mentioned above adopted “caper,” “ecodist,” “geosphere,” “ggplot2,” “ggrepel,” “ggtern,” “Hmisc,” “iCAMP,” “igraph,” “NST,” “picante,” “reshape2,” “spaa,” “stats4,” “TITAN2,” “vcd,” “vegan,” and “WGCNA” packages of R (https://www.r-project.org)^20^, ^21^, ^61^, ^62^.

## AUTHOR CONTRIBUTIONS

Yuyi Yang, Quanfa Zhang, and Wenzhi Liu conceived the idea and designed the research. Yuyi Yang and Wenzhi Liu collected experimental samples. Wenjie Wan and Peng Chen conducted whole experiments. Wenjie Wan analyzed the data and wrote the manuscript. Geoffrey M. Gadd, Ji‐Dong Gu, Quanfa Zhang, and Yuyi Yang revised the manuscript, and Yuyi Yang submitted the final version of manuscript.

## ETHICS STATEMENT

No animals and human were involved in this study.

## CONFLICT OF INTERESTS

The authors declare no conflict of interests.

## Supporting information

Supporting information.

## Data Availability

The MiSeq raw sequencing data have been deposited in the NCBI Short Read Archive database under accession numbers PRJNA860072 for archaea, PRJNA860069 for bacteria, and PRJNA860068 for fungi.
