# Supplementary material for: Beyond biogeographic patterns: Processes shaping the microbial landscape in soils and sediments along the Yangtze River
Source: mLife. 2023 Mar 26;2(1):89–100. doi: 10.1002/mlf2.12062 (PMC10989888; doi:10.1002/mlf2.12062)
Supplement: Supplementary file 1 — Supporting information. [file MLF2-2-89-s001.docx]

Supporting information for:

**Beyond biogeographic patterns: processes shaping the microbial landscape in soils and sediments along the Yangtze River**

Wenjie Wan^1, 2^, Geoffrey Michael Gadd^3, 4^, Ji-Dong Gu^5^, Wenzhi Liu^1, 2^, Peng Chen^1, 2^, Quanfa Zhang^1, 2^, Yuyi Yang^1, 2,*^

^1^Key Laboratory of Aquatic Botany and Watershed Ecology Wuhan Botanical Garden, Chinese Academy of Sciences, Wuhan 430074, PR China

^2^Danjiangkou Wetland Ecosystem Field Scientific Observation and Research Station, Chinese Academy of Sciences & Hubei Province, Wuhan 430074, PR China

^3^Geomicrobiology Group, School of Life Sciences, University of Dundee, Dundee DD1 5EH, Scotland, UK

^4^State Key Laboratory of Heavy Oil Processing, State Key Laboratory of Petroleum Pollution Control, China University of Petroleum, Beijing 102249, PR China

^5^Environmental Science and Engineering Group, Guangdong Technion-Israel Institute of Technology, 241 Daxue Road, Shantou, Guangdong 515063, PR China

*Corresponding Author

**E-mail**: [yangyy@wbgcas.cn](mailto:yangyy@wbgcas.cn) (Yuyi Yang)

**ORCID**: Yuyi Yang: <https://orcid.org/0000-0001-9807-6844>

Wenjie Wan: <https://orcid.org/0000-0001-7150-6138>

Geoffrey Michael Gadd: https://orcid.org/0000-0001-6874-870X

Address: Wuhan Botanical Garden, Lumo Road No.1, Wuchang District, Wuhan, PR China. Phone: +86-27-87700853; Fax: +86-27-87510251.

**Supplementary Method 1: Descriptions of the determination of physicochemical properties**

Soils and sediments were freeze-dried before determining physicochemical properties. Electrical conductivity (EC) and pH was determined in a 1:2.5 (w/v) soil: water suspension using a Leici conductivity meter (Shanghai, China) and a digital sartorius pH meter (Beijing, China), respectively. Total carbon (TC), total nitrogen (TN), and total sulfur (TS) were determined by using a Vario Max element analyzer (Elementar, Langenselbold, Germany). Ammonium nitrogen (NH4) and nitrate nitrogen (NO3) were obtained from K_2_SO_4_ and KCl extracts, respectively, and the content of NH_4_^+^–N and NO_3_^–^–N were measurement by using a SEALAA3 automatic flower analyzer (Seal, Germany). Soil total K (TK) and total P (TP) were heat-digested by using concentrated H_2_SO_4_-HClO_4_ and measured by flame photometry and molybdenum-blue colorimetry, respectively (Jackson, 1958). Available K (AK) was extracted using 1 mol/L NH_4_OAc (w/v, 1:10) and analyzed by atomic-absorption spectrophotometry (David, 1960). Available phosphorus (AP) was determined by extracting soil with 0.5 mol/L NaHCO_3_ (pH 8.5) at a 1: 20 soil to solution ratio; the extracted solution was then filtered through Whatman Grade No. 42 Quantitative Filter Paper (Olsen et al., 1954). Inorganic phosphorus (IP) was extracted by using 1 mol/L HCl, and the residues were digestion in 180°C for 2 h to get organic phosphorus (IP) (Waterlot, 2018). The content of IP and OP were measured by using molybdenum-blue colorimetry. Total iron (TFe) and available iron (AFe) were extracted by applying concentrated H_2_SO_4_-HClO_4_ and DTPA (diethylenetriamine pentaacetic acid)-CaCl2-TEA (triethanolamine) solution, respectively. The content of TFe and AFe were measured by using an AA240FS atomic-absorption spectrophotometer (Varian Company, USA).

**Supplementary Method 2: Estimation of environmental breadths and phylogenetic signals of microorganisms in response to environments**

Environmental breadths were defined as the threshold value of microorganisms (i.e., archaea, bacteria, and fungi) in response to environmental gradients and were calculated by applying threshold indicator taxa analysis (TITAN) in the “TITAN2” package of R (Baker and King, 2010). The sums of taxa scores for microbial ASVs were used to determine upper and lower thresholds of difference in microorganisms between soils and sediments based on environmental variables.

Before evaluating microbial phylogenetic signal to each tested environmental factor by using Fritz-Purvis *D* test (Goberna and Verdú, 2016), we firstly estimated potential trait information by calculating Spearman correlations between relative abundances of taxa and each environmental factor (Jiao and Lu, 2020; Wan et al., 2021). For example, the amplicon sequence variants (ASVs) positively or negatively correlated with temperature were regarded as “high-temperature-preferred” or “low- temperature -preferred”; the ASVs positively or negatively correlated with pH were designated as “alkaline-preferred” and “acid-preferred”. The Fritz-Purvis *D* values were calculated by using the “phylo.D” function in the “caper” package of R (Orme et al., 2013). Subsequently, the *D* value was transformed into –*D* + 1 (Goberna and Verdú, 2016; Jiao and Lu, 2020). The evolution of a trait does not reflect a noticeable signal when –*D* + 1 = 0, and is more conserved than expected by chance if –*D* + 1 > 0 (Goberna and Verdú, 2016).

**Table S1** Terrain information about sampling sites. A total of 38 sites along the Yangtze River were selected for sampling in October and November in 2019.

| Sites | Longitude (°/E) | Latitude (°/N) | Altitude  (m) | MAP  (mm) | MAT  (°C) |
| --- | --- | --- | --- | --- | --- |
| S1 | 95.75 | 34.08 | 4076.6 | 82.75 | 15.59 |
| S2 | 99.02 | 29.79 | 2440.03 | 84.5 | 15.13 |
| S3 | 96.97 | 33.35 | 3632.6 | 83.58 | 15.63 |
| S4 | 99.31 | 28.23 | 1963.14 | 84 | 15.76 |
| S5 | 100.07 | 27 | 1841 | 84.92 | 15.57 |
| S6 | 100.58 | 26.22 | 1238 | 86 | 15.28 |
| S7 | 101.86 | 26.55 | 979 | 6.25 | 22.29 |
| S8 | 102.94 | 26.9 | 634 | 124.33 | 17.12 |
| S9 | 103.89 | 28.63 | 408 | 125.67 | 16.86 |
| S10 | 104.73 | 28.78 | 278 | 117.08 | 17.36 |
| S11 | 105.62 | 28.86 | 215 | 116.5 | 17.46 |
| S12 | 106.21 | 29.21 | 189 | 97.33 | 16.28 |
| S13 | 106.72 | 29.56 | 182 | 87.5 | 15.8 |
| S14 | 107.48 | 29.83 | 178 | 115.25 | 16.51 |
| S15 | 108.44 | 30.85 | 159 | 107.5 | 16.32 |
| S16 | 111.32 | 30.65 | 46 | 101.67 | 16.98 |
| S17 | 112.26 | 30.21 | 50 | 106.67 | 17.27 |
| S18 | 112.9 | 29.73 | 34 | 89.5 | 16.98 |
| S19 | 113.51 | 29.83 | 28 | 91.83 | 16.56 |
| S20 | 114.08 | 30.21 | 6 | 111.33 | 17.04 |
| S21 | 114.53 | 30.66 | 6 | 117.33 | 17.22 |
| S22 | 114.96 | 30.4 | 21 | 105.67 | 17.1 |
| S23 | 115.13 | 30.22 | 18 | 104 | 16.96 |
| S24 | 115.59 | 29.83 | 13 | 94.75 | 18.2 |
| S25 | 116.03 | 29.75 | 12 | 91.58 | 16.5 |
| S26 | 116.59 | 29.96 | 13 | 69.42 | 19.04 |
| S27 | 117.02 | 30.49 | 14 | 98.67 | 17.77 |
| S28 | 117.69 | 30.78 | 10 | 100.25 | 18.49 |
| S29 | 117.92 | 31.08 | 7 | 105.33 | 18.13 |
| S30 | 118.33 | 31.37 | 12 | 91.92 | 18.63 |
| S31 | 118.57 | 31.89 | 33 | 94.08 | 18.54 |
| S32 | 118.98 | 32.19 | 6 | 54.92 | 9.62 |
| S33 | 119.57 | 32.2 | 4 | 38.25 | 2.66 |
| S34 | 119.96 | 32 | 15 | 34.75 | -1.92 |
| S35 | 120.93 | 31.88 | 6.65 | 68.5 | 20.5 |
| S36 | 121.67 | 31.73 | 10.05 | 64 | 21.15 |
| S37 | 121.76 | 31.47 | 9.48 | 74.75 | 13.31 |
| S38 | 121.59 | 31.43 | 4 | 72.75 | 14.68 |

Abbreviations: MAP, mean annual precipitation; MAT, mean annual temperature.

**Table S2** PERMANOVA showing effects of environmental variables on community composition of microorganisms (i.e., archaea, bacteria, and fungi) in soils and sediments. Abbreviations of environmental variables are defined in “Materials and methods” section. Asterisks denote significance (*, *p* < 0.05; **, *p* < 0.01; ***, *p* < 0.001).

| Factor | Archaeal community | | Bacterial community | | Fungal community | |
| --- | --- | --- | --- | --- | --- | --- |
|  | Soil | Sediment | Soil | Sediment | Soil | Sediment |
| Lon | 13.95%*** | 14.95%*** | 10.68%*** | 11.10%*** | 2.38%* | 2.74%* |
| Lat | 5.20%*** | 5.12%*** | 5.60%*** | 4.00%*** | 2.50%** | 2.31% |
| Alt | 11.93%*** | 12.38%*** | 8.02%*** | 10.21%*** | 1.95% | 1.32% |
| MAP | 1.91% | 1.46% | 2.20%* | 1.74% | 1.93% | 1.40% |
| MAT | 2.28%* | 1.13% | 3.02%** | 1.24% | 2.43%* | 1.41% |
| pH | 4.20%*** | 6.05%*** | 2.56%** | 5.65%*** | 2.62%* | 3.00%* |
| EC | 2.90%** | 3.96%*** | 2.98%** | 4.07%*** | 2.00% | 2.11% |
| TC | 2.52%* | 3.18%** | 2.77%** | 3.14%*** | 1.98% | 3.28%** |
| TN | 2.61%* | 4.32%*** | 2.82%** | 4.54%*** | 2.66%* | 6.32%*** |
| NH_4_^+^–N | 4.12%** | 5.98%*** | 3.55%*** | 5.01%*** | 2.22%* | 2.88%* |
| NO_3_^–^–N | 3.30%** | 1.69% | 3.14%** | 1.83% | 2.43%* | 1.84% |
| TP | 2.76%* | 4.25%*** | 2.44%** | 4.31%*** | 2.21%* | 2.06% |
| AP | 5.21%*** | 4.71%*** | 4.30%*** | 4.24%*** | 1.69% | 1.34% |
| IP | 3.10%** | 4.53%*** | 2.58%** | 4.30%*** | 2.30%* | 1.48% |
| OP | 1.85% | 1.50% | 1.89% | 1.91% | 1.76% | 1.63% |
| TS | 2.57%* | 2.47%* | 3.15%** | 2.73%** | 2.34%* | 1.32% |
| TK | 6.41%*** | 4.43%*** | 5.13%*** | 4.01%*** | 2.32%* | 2.52%* |
| AK | 3.65%*** | 4.32%*** | 3.74%*** | 3.70%*** | 1.80% | 1.84% |
| TFe | 2.53%* | 2.41%* | 2.78%** | 2.98%** | 1.77% | 1.11% |
| AFe | 12.59%*** | 5.60%*** | 9.76%*** | 4.95%*** | 2.92%** | 2.43%* |

**Table S3** Pearson’s correlations between environmental factors and community diversities (Shannon-Wiener index) of microorganisms (i.e., archaea, bacteria, and fungi) in soils and sediments. Abbreviations of environmental variables are defined in “Materials and methods” section. Asterisks denote significance (*, *p* < 0.05; **, *p* < 0.01; ***, *p* < 0.001).

| Factor | Archaeal community | | Bacterial community | | Fungal community | |
| --- | --- | --- | --- | --- | --- | --- |
|  | Soil | Sediment | Soil | Sediment | Soil | Sediment |
| Lon | 0.578*** | 0.648*** | 0.348** | 0.297** | -0.224* | -0.297* |
| Lat | 0.169 | 0.172 | 0.143 | 0.116 | -0.058 | -0.250* |
| Alt | -0.515*** | -0.540*** | -0.346** | -0.269* | -0.138 | 0.162 |
| MAP | -0.029 | -0.097 | 0.167 | 0.068 | 0.194 | -0.012 |
| MAT | 0.078 | 0.005 | 0.008 | 0.168 | 0.059 | 0.161 |
| pH | -0.030 | -0.190 | -0.077 | -0.087 | -0.104 | -0.018 |
| EC | -0.137 | -0.025 | -0.284* | -0.276* | -0.200 | -0.048 |
| TC | -0.272* | -0.119 | 0.040 | -0.087 | 0.175 | 0.091 |
| TN | -0.156 | -0.067 | 0.096 | -0.097 | 0.051 | 0.042 |
| NH_4_^+^–N | 0.002 | 0.367*** | 0.203 | 0.128 | -0.076 | -0.206 |
| NO_3_^–^–N | -0.386*** | -0.146 | -0.075 | 0.096 | 0.027 | 0.136 |
| TP | -0.020 | 0.325** | 0.203 | 0.144 | -0.076 | 0.105 |
| AP | 0.289* | 0.306** | 0.323*** | 0.110 | 0.250* | 0.023 |
| IP | 0.084 | 0.461*** | 0.274* | 0.248* | 0.311** | -0.006 |
| OP | -0.099 | -0.082 | 0.126 | -0.094 | 0.019 | 0.197 |
| TS | -0.295** | -0.088 | -0.050 | -0.207 | 0.140 | 0.111 |
| TK | 0.163 | 0.149 | 0.288* | 0.008 | -0.014 | -0.018 |
| AK | -0.181 | 0.161 | -0.240* | 0.008 | -0.274* | -0.050 |
| TFe | -0.139 | 0.038 | -0.237* | -0.022 | -0.319** | 0.051 |
| AFe | 0.407*** | 0.388*** | 0.327*** | 0.144 | 0.095 | -0.115 |

**Table S4** Environmental breadths and phylogenetic signals of microorganisms in response to each tested factors.

| Groups | Specific environmental factors | Tested factors | Ratio |
| --- | --- | --- | --- |
|  | Environmental breadths | | |
| Archaea-Soil | 14 (i.e., Lon, Lat, Alt, MAP, MAT, EC, TC, TN, TP, AP, OP, TS, TK, and TFe) | 20 | 14/20*100%=70% |
| Archaea-Sediment | 6 (i.e., pH, NH_4_^+^–N, NO_3_^–^–N, IP, AK, and AFe) | 20 | 6/20*100%=30% |
| Bacteria-Soil | 13 (i.e., Lon, Lat, Alt, MAP, MAT, pH, TC, TN, NO_3_^–^–N, AP, IP, OP, and TK) | 20 | 13/20*100%=65% |
| Bacteria-Sediment | 7 (i.e., EC, NH_4_^+^–N, TP, TS, AK, TFe, and AFe) | 20 | 7/20*100%=35% |
| Fungi-Soil | 14 (i.e., Lat, MAP, MAT, pH, EC, TN, NO_3_^–^–N, TP, AP, IP, OP, TS, TK, and TFe) | 20 | 14/20*100%=70% |
| Fungi-Sediment | 6 (i.e., Lon, Alt, TC, NH_4_^+^–N, AK, and AFe) | 20 | 6/20*100%=30% |
|  |  |  |  |
|  | Phylogenetic signals | | |
| Archaea-Soil | 15 (i.e., MAP, MAT, pH, EC, TN, NH_4_^+^–N, NO_3_^–^–N, TP, AP, IP, OP, TK, AK, TFe, AFe) | 20 | 15/20*100%=75% |
| Archaea-Sediment | 5 (i.e., Lon, Lat, Alt, TC, and TS) | 20 | 5/20*100%=25% |
| Bacteria-Soil | 14 (i.e., Lon, Lat, MAP, MAT, pH, EC, TC, TN, NO_3_^–^–N, TP, AP, OP, AK, and AFe) | 20 | 14/20*100%=70% |
| Bacteria-Sediment | 6 (i.e., Alt, NH_4_^+^–N, IP, TS, TK, and TFe) | 20 | 6/20*100%=30% |
| Fungi-Soil | 15 (i.e., Lon, Lat, Alt, MAP, MAT, EC, NH_4_^+^–N, NO_3_^–^–N, AP, IP, OP, TS, TK, AK, AFe) | 20 | 15/20*100%=75% |
| Fungi-Sediment | 5 (i.e., pH, TC, TN, TP, and TFe) | 20 | 5/20*100%=25% |

Note: The numbers of tested environmental factors is 20 (i.e., Lon, Lat, Alt, MAP, MAT, pH, EC, TC, TN, NH_4_^+^–N, NO_3_^–^–N, TP, AP, IP, OP, TK, AK, TFe, and AFe). Broader environmental breadths and stronger phylogenetic signals of microorganisms were named as “specific environmental factors”. Abbreviations are defined in “Materials and methods” section.

**Table S5** Pearson’s correlations between logitude and physicochemical properties for soils and sediments. Abbreviations of environmental variables are defined in “Materials and methods” section. Asterisks denote significance (*, *p* < 0.05; **, *p* < 0.01; ***, *p* < 0.001).

| Factors | Soil | Sediement |
| --- | --- | --- |
| pH | -0.290* | -0.479*** |
| EC | 0.241* | 0.199 |
| TC | -0.249* | -0.341** |
| TN | 0.103 | 0.058 |
| NH_4_^+^–N | 0.226* | 0.490*** |
| NO_3_^–^–N | -0.320** | -0.084 |
| TP | 0.127 | 0.187 |
| AP | 0.350** | 0.446*** |
| IP | 0.167 | 0.252* |
| OP | 0.051 | -0.032 |
| TS | -0.385*** | -0.161 |
| TK | 0.383*** | 0.369*** |
| AK | 0.317** | 0.381*** |
| TFe | 0.238* | -0.191 |
| AFe | 0.645*** | 0.429*** |


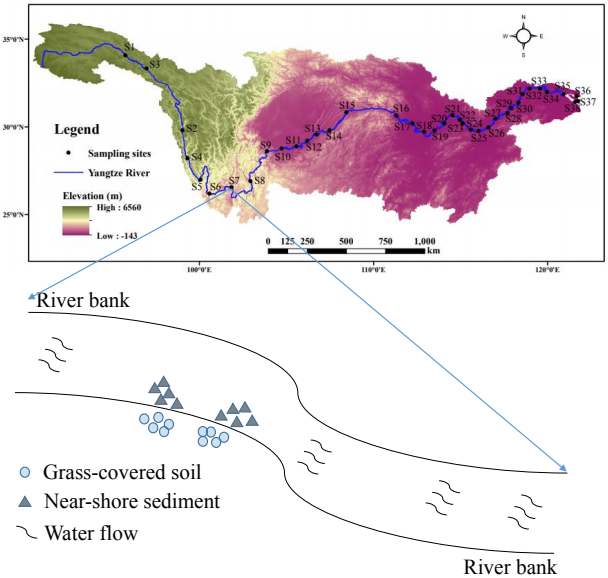


**Figure S1**. The locations of 38 sampling sites and sampling details. Five soil or sediment cores were collected at a depth of 0–20 cm with a hand core probe at each site. Soil or sediment cores were then mixed evenly to form a composite soil or sediment sample.


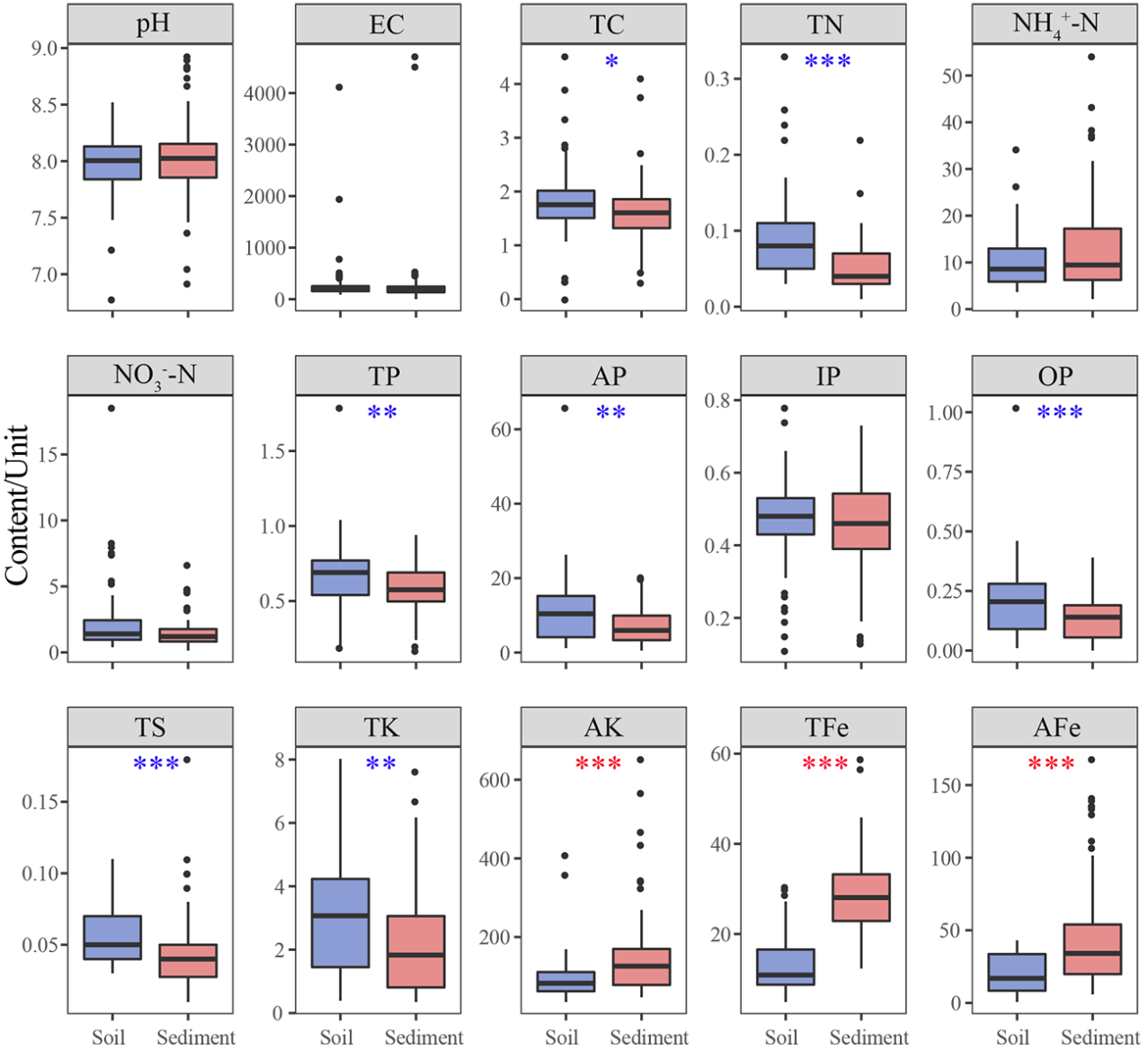


**Figure S2**. Differences in physicochemical properties between soils and sediments. Asterisk denotes significance (*, *p* < 0.05). Abbreviations: EC, electrical conductivity; TC, total carbon; TN, total nitrogen; NH4, ammonia nitrogen; NO3, nitrate nitrogen; TP, total phosphorus; AP, available phosphorus; IP, inorganic phosphorus; OP, organic phosphorus; TS, total sulfate; TK, total potassium; AK, available potassium; TFe, total iron; and AFe, available iron.


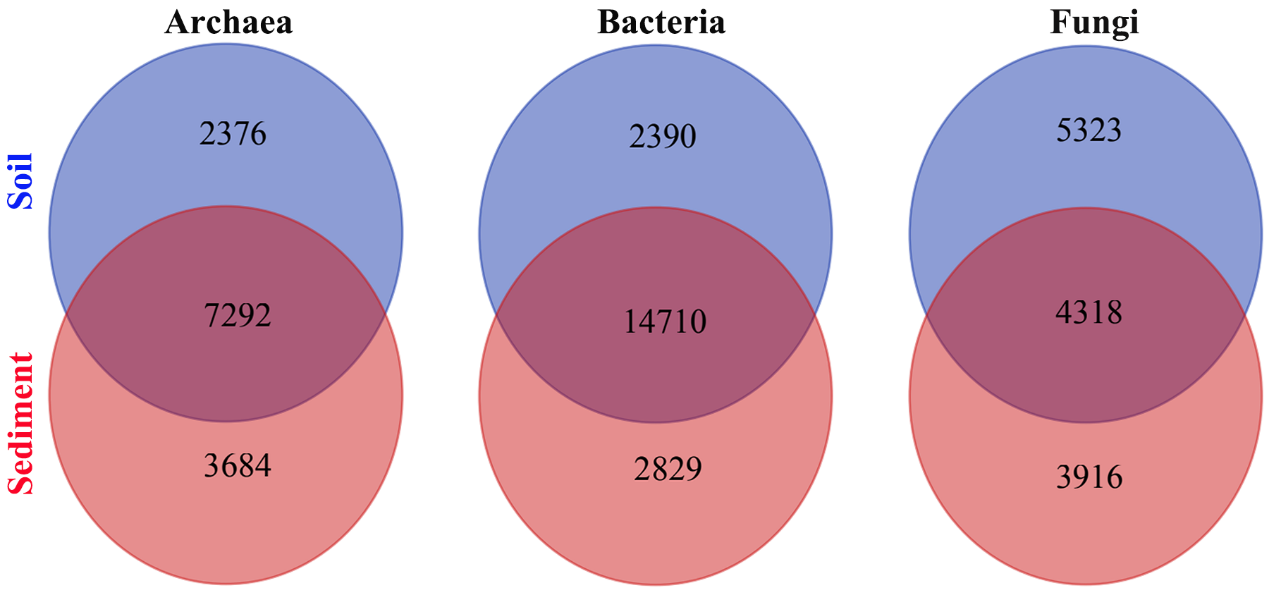


**Figure S3**. Veen diagram showing shared ASVs of microorganisms (i.e., archaea, bacteria, and fungi) between soils and sediments.


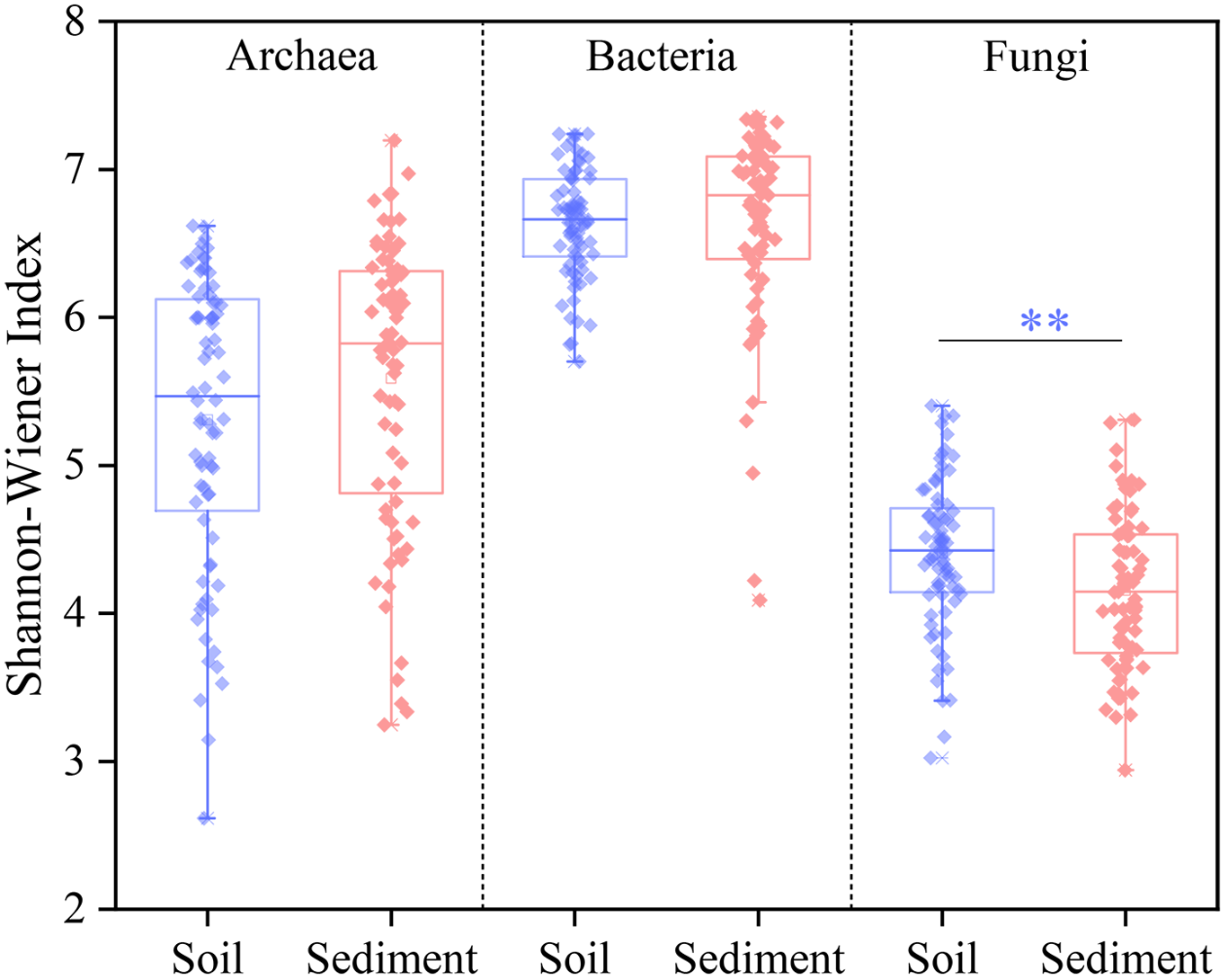


**Figure S4**. Differences in Shannon-Wiener index of microorganisms (i.e., archaea, bacteria, and fungi) between soils and sediments. Asterisks denote significance (**, *p* < 0.01).

**References**

Baker ME, King RS. A new method for detecting and interpreting biodiversity and ecological community thresholds. *Methods Ecol Evol*. 2010;1:25–37.

David DJ. The determination of exchangeable sodium, potassium, calcium and magnesium in soils by atomic-absorption spectrophotometry. *Analyst*. 1960;85:495–503.

Goberna M, Verdú M. Predicting microbial traits with phylogenies. *ISME J*. 2016;10:959–967.

Jackson ML. Soil Chemical Analysis. Prentice-Hall, Inc., Englewood Cliffs, NJ. 1958.

Jiao S, Lu Y. Abundant fungi adapt to broader environmental gradients than rare fungi in agricultural fields. *Global Change Biol*. 2020;26:4506–4520.

Olsen SR, Cole CV, Watanbe FS, Dean LA. Estimation of Available Phosphorus in Soils by Extraction With Sodium Bicarbonate. Circ No 939. USDA, Washington D.C. 1954.

Orme D, Freckleton R, Thomas G, Petzoldt T, Fritz SA, Isaac N, et al. Caper: comparative analyses of phylogenetics and evolution in R. *Methods Ecol Evol*. 2013;3:145–151.

Wan W, Gadd GM, Yang Y, Yuan W, Gu J, Ye L, et al. Environmental adaptation is stronger for abundant rather than rare microorganisms in wetland soils from the Qinghai-Tibet Plateau. *Mol Ecol.* 2021;30:2390–2403.

Waterlot C. Alternative approach to the standard, measurements and testing program used to establish phosphorus fractionation in soils. *Analyt Clim Acta*. 2018;1003:26–33.
